# Nanoimprinted Hybrid Metal-Semiconductor Plasmonic Multilayers with Controlled Surface Nano Architecture for Applications in NIR Detectors

**DOI:** 10.3390/ma8085028

**Published:** 2015-08-07

**Authors:** Akram A. Khosroabadi, Palash Gangopadhyay, Steven Hernandez, Kyungjo Kim, Nasser Peyghambarian, Robert A. Norwood

**Affiliations:** College of Optical Sciences, the University of Arizona, Tucson 85721, AZ, USA; E-Mails: aamooali@email.arizona.edu (A.A.K.); sahernandez2@gmail.com (S.H.); kyungjokim@email.arizona.edu (K.K.); nasser@optics.arizona.edu (N.P.); rnorwood@optics.arizona.edu (R.A.N.)

**Keywords:** surface plasmon, metal semiconductor interface, silicon, photodetectors, infrared

## Abstract

We present a proof of concept for tunable plasmon resonance frequencies in a core shell nano-architectured hybrid metal-semiconductor multilayer structure, with Ag as the active shell and ITO as the dielectric modulation media. Our method relies on the collective change in the dielectric function within the metal semiconductor interface to control the surface. Here we report fabrication and optical spectroscopy studies of large-area, nanostructured, hybrid silver and indium tin oxide (ITO) structures, with feature sizes below 100 nm and a controlled surface architecture. The optical and electrical properties of these core shell electrodes, including the surface plasmon frequency, can be tuned by suitably changing the order and thickness of the dielectric layers. By varying the dimensions of the nanopillars, the surface plasmon wavelength of the nanopillar Ag can be tuned from 650 to 690 nm. Adding layers of ITO to the structure further shifts the resonance wavelength toward the IR region and, depending on the sequence and thickness of the layers within the structure, we show that such structures can be applied in sensing devices including enhancing silicon as a photodetection material.

## 1. Introduction

Surface plasmon resonance (SPR) refers to a collective, but localized, oscillation of the conduction electrons that appear at sharp features (corners or edges) in metallic nanostructures when excited by an electromagnetic (EM) field [1,2,3]. Their optical properties are determined by the permittivities of both the metal and the surrounding dielectric, as well as by the morphology of the nanostructure [4]. By tuning the surrounding dielectric medium intriguing plasmonic structures have been reported for broad-spectrum plasmon enhanced applications. Hybrid metal/dielectric nanostructures have been used to concentrate and channel light using subwavelength structures [5], to enhance the signal in surface-enhanced Raman spectroscopy (SERS) [6], to carry information in microprocessors faster than current transistors [7], and to encode much more information than is currently possible with conventional electronics [8,9]. However, when a semiconductor material with relatively smaller optical cross-section is used as the surrounding dielectric, the plasmons go through periodic convective (within the metal) and diffusive (within the semiconductor) transport, resulting in a wider spatial distribution of the oscillation [10]. Upon optical excitation, nonlocal surface plasmons (SPs) propagate along the interface of a metal semiconductor hybrid structure like ripples across the surface of water. Nonlocalization of SPs has been the key reason hybrid nanostructures composed of semiconductor transparent oxides and plasmonic metal components are receiving extensive attention. They display extraordinary optical characteristics that are derived from the simultaneous existence and close coupling of localized surface plasmon resonance and semiconductor optoelectronic properties, as well as the synergistic interactions between the two components. Nonlocal SPs have been suggested as the driving physics in the enhanced visible light catalytic response of TiO_2_ nanostructures when integrated with plasmonic metals [11,12,13,14,15]. A number of different types of hybrid plasmonic metal semiconductor structures have been designed and synthesized [16,17] for applications such as plasmon enhanced photo catalytic reactions [13,18,19], photoluminescence [20,21], solar cells [17,22], and various biotechnological applications [23,24,25].

Silicon photodetectors have been the mainstay for efficient detection within the visible range of the spectrum over many decades. The mature CMOS technology allows fabrication of such detectors over large areas and in large quantities. However, outside the visible range of the spectrum, in the near infrared (NIR) or infrared (IR) regimes, such detectors fail to operate, as the incident photons are lower in energy and are unable to excite electrons from the valence band to the conduction band in Si detectors. For operation within the NIR and IR regimes, the most popular choices have been either germanium (Ge) or indium gallium arsenide (InGaAs) detectors and both platforms have been integrated with silicon photonics technology [26,27]. Despite these developments, an all-silicon solution for NIR and IR photodetection in silicon photonics technology is still drawing much attention. Several CMOS process-compatible approaches were proposed and demonstrated, including two-photon absorption, insertion of mid-band gap defect states into the silicon lattice, cavity enhanced photocurrent generation, internal photoemission (IPE) process, and integration of a germanium active layer with the silicon-based device [28,29,30]. Among these, the IPE process has been the most promising, where the Schottky barrier created at the interface of Si and a metal (such as, Cu, Al, Ag and Au) is typically lower in energy than the band gap of Si, thereby facilitating the crossover of lower energy photon-generated electrons. Assuming that the photon energy is higher than the Schottky barrier, these electrons can cross over the barrier into the doped Si where they can be collected as a photocurrent under a reverse bias. However, the efficiency of this process is low due to momentum mismatch, low volume of interaction between the incoming photons and the electrons, and, in particular, lack of broadband absorption from the metal. In recent years, several different configurations have been explored for efficient IPE with the help of localized surface plasmon resonance [31,32,33,34,35]. While improvement in efficiency, responsivity, and absorption enhancement have been shown, there is still need for a better Si detector in the NIR and IR regime that offers better quantum efficiency over a broad spectral range within the telecommunication wavelength range.

In this paper we have fabricated and explored several alternative designs where a transparent conducting oxide layer, in particular, indium tin oxide (ITO), has been added underneath the plasmonic metal Ag to achieve nonlocal behavior of the plasmon resonance and enhanced broadband absorption. Although a working photodiode was not built, various simulated photodetector designs were built by coating Si on multilayer Ag-ITO nanostructures deposited on a high aspect ratio polyacrylonitrile nanoimprinted scaffold. By changing the dimensions and thicknesses of various layers, we show that the plasmon resonance can be tuned to achieve enhanced broadband absorption in the NIR and IR regimes. This is of particular interest for wavelengths over 1150 nm, the maximum operational wavelength for current Si avalanche photodiodes. We also use FDTD simulations to probe the efficacy of these designs to control and achieve nonlocality in the surface plasmon resonance in the Ag layer.

## 2. Results and Discussion

### 2.1. Electrode Fabrication

**Figure 1:**
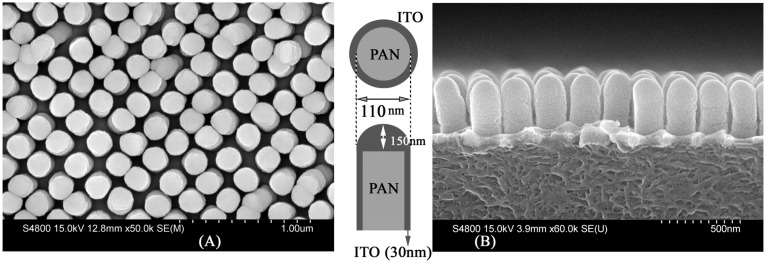
(**A**) top view and (**B**) side view SEM of ITO nanopillar array, *inset* shows the construction and dimensions of individual pillars.

Fabrication of imprinted nanopillar structures of polyacrylonitrile (PAN) with the desired pitch/aspect ratio and their characterization has been previously reported [6,36,37,38]. 1 cm × 1 cm ITO nanopillar electrodes were fabricated by depositing ITO on the previously nanoimprinted PAN structures, using an electron beam deposition technique. The nanopillars have an average diameter of 170 ± 8.8 nm and height of 442 ± 10.6 nm; the error is the width at half maximum in the Gaussian distribution profile computed from scanning electron micrographs (SEM) using ImageJ [39]. The center-to-center pitch in these structures was 200 ± 2.4 nm resulting in an average wall-to-wall distance of ~15 nm. A large area top view and cross-sectional SEM of nanostructured ITO (nsITO) is shown in Figure 1. This structure was fabricated by depositing 150 nm ITO onto PAN nanopillars resulting in a 30 nm thick coating on the sidewalls and a 150 nm coating on top of the pillars and in the planar region at the base of the pillars. In a similar fashion, by sequential deposition of ITO and Ag, multiple nanopillar array structures were fabricated in layer configurations AB, BA, ABA, and BAB, where A and B are ITO and Ag, respectively.

### 2.2. Discussion

The surface area in the nsITO shown in Figure 1A is 5.6 times larger than that of the planar ITO with 180 nm thickness and this is mostly due to the relatively thinner (~30 nm) sidewall region of the nanopillars. Thickness-dependent band gap modifications in deposited ITO films, previously reported by the authors [38] and others [40,41], suggest that quantum confinement of the free carriers in ITO is expected within the 30 nm thick shell, contributing significantly to the overall optical and electrical properties. The nsITO is more transparent across the entire spectrum and shows lower specular reflection. The band edge of the nsITO is red shifted and shows a second smaller optical band gap ~3.25 eV in addition to a bulk optical band gap at 3.55 eV. Bulk optical band gaps of 3.4 to 3.7 eV have been reported in literature. Recently we have shown that the optical band gap can be fine-tuned by changing the shell thickness on the sidewall [38]. nsITO also shows lower specular reflection and the carrier concentration is ~5–6 times larger than for the planar sample; this is also a strong function of the shell thickness. The resistivity of a 1 cm × 1 cm area for nsITO is higher than that of the planar equivalent, but scales with the surface area enhancement factors, indicating that an actual device using these nsITOs could be prepared with smaller active areas. In devices such as organic solar cells, the nanostructured electrodes indirectly organize the active material into nanoscale features with dimensions close to the exciton diffusion lengths of common bulk heterojunction materials [42,43].

To further investigate the role of the core and embedding media on the optical properties of the nanostructured electrodes we developed nanopillar arrays of electrodes with ITO and Ag core shell type configuration in different orders. The optical characteristics of these electrodes are strong functions of the geometry of the pillars and surrounding dielectrics and can be explained using traditional core-shell plasmonic models based on plasmon hybridization [44]. Detailed modeling and characterization indicate that by changing the core and embedding dielectrics optical and electrical properties can be modified.

**Figure 2:**
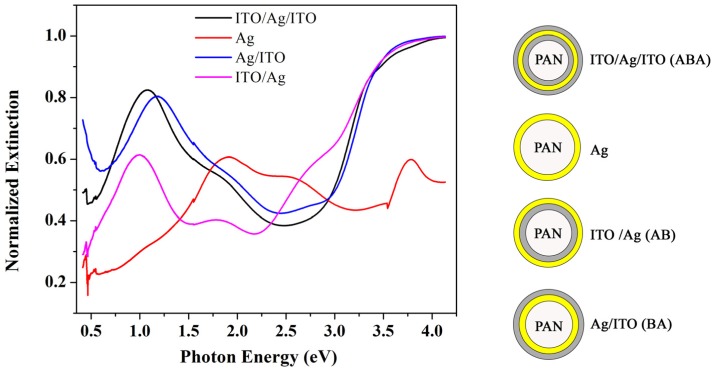
Extinction spectra of multilayer core shell nanostructured electrodes.

Figure 2 shows extinction spectra of different multilayer core shell electrodes. Extinction spectra within these structures point towards a highly tunable plasmon response by changing the core and surrounding shell medium properties. For comparison, we have also plotted the extinction spectrum of 10 nm Ag deposited on PAN pillars. There are different local maxima in the extinction spectrum of Ag around 492 nm and 690 nm. We attribute this to surface plasmon absorption in the Ag nanoparticle and a hybridized plasmon resonance, respectively. The hybridized resonance frequency tends to red shift with increased Ag thickness; this property can be used in solar cells or detectors to increase light absorption in the active layer by tuning the plasmon frequency accordingly [45]. The surface plasmon frequency red shifts significantly by adding layers of ITO, which is a direct consequence of plasmon hybridization as will be discussed in detail below.

One of the interesting properties of these multilayer core shell type structures is the difference in their surface resistivity measured using the four-probe technique. As mentioned earlier, the resistivity of nsITO is higher than for the planar sample and depends on the surface area, but by adding a thin layer of Ag underneath the ITO layer, the resistivity can be decreased from 1800 to 65 Ω/□. Table 1 shows resistivity values for different planar and nanopillar arrays. As expected, with increasing the conductivity, transmission decreases.

**Table 1:** Electrical and optical characteristics of different core shell electrodes.

Structure	Thickness (nm)	Resistivity (Ω/□)		Optical band gap (eV)	
		nanostructured	planar	nanostructured	bulk
ITO/Ag	150/10	1800	10–15	2.75	3.22
Ag/ITO	10/150	65	15	-	-
ITO/Ag/ITO	70/10/70	150	7	2.79	3.14
ITO	180	2000	85	2.92	3.2

The optical band gaps of different core shell nanostructures were calculated using the Tauc relation [46]. A detailed discussion of the band gap calculation and its dependence on the dimensions of the nanopillars are given elsewhere [38]. Band gap changes are attributed to nanostructure morphology, defects and surface microstructures [47]. Along with the bulk optical band gap, a lower energy band gap is also obtained, which usually comes from generated impurity levels in the conduction band. According to the Burstein-Moss effect, the bulk band gap increases with increasing free carrier concentration, leading to a blue shift of the absorption edge [48]. Both bulk and nanostructure band gap values for different nanopillar arrays are also shown in Table 1. The change in the optical band gap of the electrodes upon altering the sequence of the layers could be because of strain induced by change in the grain size of different layers and many body interactions [49]. Indium tin oxide is a highly doped wide band gap material; with increasing impurity levels, free carrier concentration is increased, higher levels in the conduction band are filled and the Fermi level is pushed to higher energies, which causes optical band gap widening; however, in highly doped semiconductors, impurity levels are created that lie at lower energies and this effect leads to band gap shrinkage [50]. The nanostructured TCOs in our study showed both smaller and larger band gaps compared to their planar counterparts.

**Figure 3:**
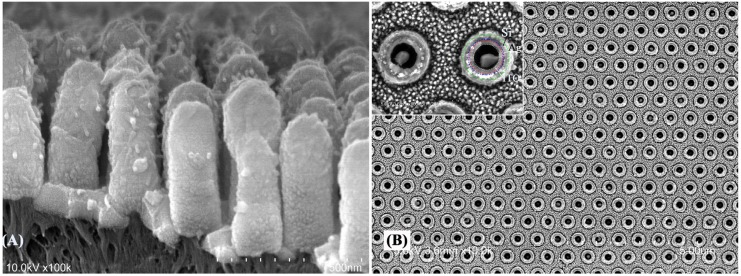
(**A**) SEM images of PAN/ITO/Ag nanopillars and (**B**) top view of etched PAN/Ag/ITO/Si multilayer electrodes.

High-resolution SEM images of two different core shell electrodes with ITO, as either core or embedding medium, are shown in Figure 3. Figure 3A shows small silver nanoparticles decorating the ITO pillars, a result of 10 nm of Ag deposited on ITO. Figure 3B shows an etched view of Ag nanoshells with ITO as embedding medium. 50 nm Si is then deposited on top of the pillars. As will be discussed below, adding ITO as the embedding or core medium shifts the resonance frequency to the IR, which makes the structures suitable for sensing applications in this region. The core shell type shape of the arrays can be seen in this image as the rings of different layers are shown and confirmed by energy dispersive x-ray spectroscopy (EDS).

### 2.3. Surface Plasmon Polariton (SPP)

A surface plasmon is a collective oscillation of free electrons at the surface of a metal at a metal/dielectric interface. The electric field enhancement at the interface depends on the difference between the dielectric constants of the metal and the dielectric medium. According to the Maxwell equations and their boundary conditions, the electric field should go to zero at the boundary between the dielectric medium and the metal. By solving the Maxwell equations with appropriate boundary conditions, the surface plasmon wave vector is expressed as

**Figure 4:**
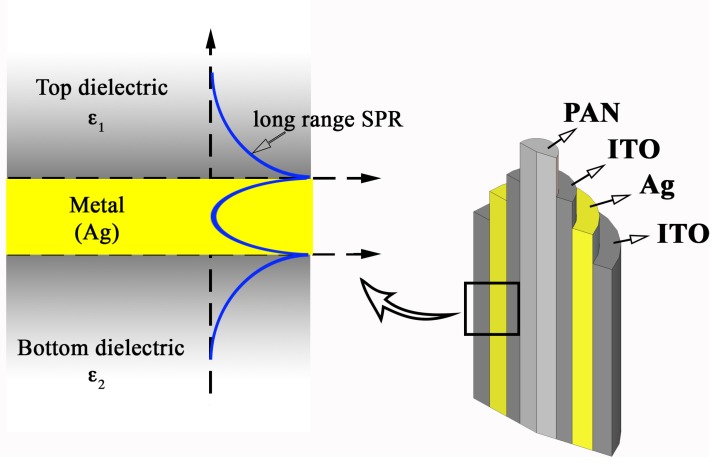
The decay of a surface plasmon excitation at the double metal semiconductor interface and generation of long range SPR within hybrid nanostructures.

(1) K(ω)=K'(ω)+iK"(ω)=[ωc(ε1'ε2ε1'+ε2)12]+i[ωc(ε1'ε2ε1'+ε2)32ε1"2(ε1')2]

with *ω* the frequency of the incident is light and *ε*_1,2_ are the dielectric constants of the metal and dielectric medium, respectively [51]. The plasmon propagation length is given by *L_SPP_*, with *L_SPP_* = 1/2*Kʺ.* Here *Kʺ* is the imaginary part of the wave vector and *L_SPP_* is the distance where the SPP intensity has decayed to 1/e of its original value. Since loss in the metal is high, the plasmon propagation length inside the metal will be short and within the metal the plasmon decays rapidly [51] (see Figure 4). In a layered plasmonic structure, when the dielectric constants of the two surrounding media are the same (*ε*_1_ = *ε*_2_), long range surface plasmon (LRSP) generation within the sandwiched plasmonic metal is primarily facilitated. LRSPs possess longer surface propagation lengths, higher electric field strengths, and sharper angular resonance curves than conventional SPs [52].

Transparent conductive oxides have recently gained tremendous interest due to their lower loss compared to noble metals, especially Au which has a large interband transition in the visible and near infrared regions [53]. The free carrier concentration of TCOs can be tuned under suitable doping and fabrication conditions by depositing a very thin layer of a noble metal (here Ag), with the effective metal carrier concentration variable through nanostructuring. The Maxwell-Garnett effective medium approximation was used to find the ratio of Ag and ITO in the core shell structures [37]. The bulk plasma frequency of Ag is 9.1 eV [54], however, depolarization charges due to the dielectric core cause a screened plasma frequency which is smaller than the bulk plasma frequency [55]. Using small nanoparticles of noble metals and nanostructuring can reduce free carrier concentration in the metal, which is comparable to that of ITO, and acts as a continuous ITO layer [56]. Lower loss in ITO induces a longer propagation distance and non-localized surface plasmons. FDTD simulations show a broader spread of hot spots in these structures and will be discussed later in the text.

### 2.4. Plasmon Hybridization Model

The plasmon hybridization model is a powerful tool for investigating plasmonic behavior of core-shell particles and can be used to explain the influence of core shell nanostructure geometry and composition on the surface plasmon frequency. The bonding and antibonding plasmon modes in different layers induce hybridization frequencies that are dependent on the layer thicknesses and properties. Different charge densities on the inner and outer interfaces of the layers will result in bonding (lower frequency) and antibonding (higher frequency) plasmon modes [57]. The bonding plasmon frequency is due to symmetric coupling between plasmons of the cavity and the particle. In Mie theory sphere (*ω_sl_*) and cavity (*ω_cl_*), plasmon frequencies for a nanoshell with a vacuum core in vacuum are given by

(2) $$\omega_{sl}=\omega_{B}\sqrt{\frac{l}{2l+1}}\;\text{and}\;\omega_{cl}=\omega_{B}\sqrt{\frac{l+1}{2l+1}}$$

*l* is the multipolar index, and is equal to 1 for dipoles; while *ω_B_* is the bulk plasma frequency of the metal, derived using the Drude model [58]. According to Drude, the bulk plasma frequency of a metal is given by

(3) $$\omega_{B}=\sqrt{\frac{4\pi e^{2}n_{0}}{m_{e}}}$$

Here *m_e_* is the effective mass of the electron and *n_o_* refers to the free carrier concentration in the metal. If the coupling between the surfaces charges of the cavity and sphere plasmons is anti-symmetric, the resulting plasmon mode will be an antibonding plasmon which lies in a higher frequency range than the cavity and particle plasmons. The hybridization frequencies are highly sensitive to the thicknesses of both the core and/or the shell surrounding the metal layer. Using the kinetic energy of the conduction electrons in the metals and the Coulomb potential energy of the induced surface charges one can calculate bonding and antibonding plasma frequencies [58]. In a spherical geometry

(4) $$\omega_{\pm }^{2}=\frac{\omega_{B}^{2}}{2}\left[1\pm \frac{1}{2l+1}\sqrt{1+4l\left(l+1\right)x^{2l+1}}\right]$$

where *x* is the ratio of the inner radius to the outer radius of the nanoshell. This equation provides the plasmon hybridization frequencies of a metallic nanoshell embedded in air. If the embedding medium changes, the dielectric constant of the medium also needs to be taken into account. Inclusion of a dielectric in the core of the nanoparticles or surrounding medium induces screening charges on the metal-dielectric surfaces that are polarization charges due to interaction of the electric field and the dielectric medium. In this case, the plasmon frequencies squared are a linear combination of the particle and cavity plasmon frequencies.

**Figure 5:**
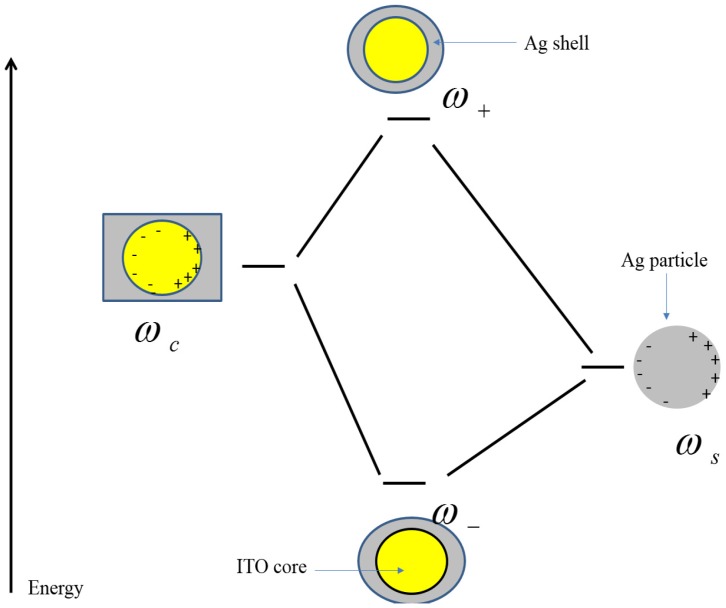
Plasmon hybridization in metallic nanoshells consisting of bonding and antibonding frequencies that are hybridized frequencies of sphere and cavity plasmons.

Figure 5 shows the energy diagram of the plasma hybridization an Ag nanoshell. As one can see, the cavity plasmon resonance frequency is much higher than that of the particle. Changes in the induced charges on the inner and outer surfaces of the shell lead to a shift of plasma frequencies compared to particle and cavity frequencies [59].

In this geometry, the small difference between inner and outer radii results in a strong interaction between the sphere and cavity plasmons and large blue and red shifts in the bonding and antibonding plasma frequencies with respect to individual plasmons is observed [60].

### 2.5. Surface Plasmon Excitation in Ag Nanopillars

Deposition of a very thin layer of Ag on the pillars creates a non-continuous film of small particles of Ag. The fundamental plasma frequency of Ag nanoparticles changes with size of the particles. Small Ag nanoparticles on a glass substrate can be fabricated using RF sputtering and, depending on the size of the nanoparticles, the surface plasmon frequency can be tuned. For example, depositing 10 nm Ag on glass results in an SPR wavelength of 454 nm [61]. One can see that the Figure 2 extinction spectrum of 10 nm Ag on the pillars shows a local maximum at around 2.52 eV (490 nm), the plasma frequency of Ag nanoparticles. In a thin metallic nanoshell of Ag, the particle can be considered as a cavity inside a metal and a metallic sphere. Aforementioned formulations are for a metallic shell in a cavity with a dielectric constant of 1. If we assume *ω_B_* = 7.8 eV for *l* = 1, cavity and sphere plasmons will be *ω_c_* = 6.21 eV and *ω_s_* = 4.51 eV. However, in the present work, the cavity core is PAN with a dielectric constant of *ε_c_* = 2.2, therefore *ω_c_* = 5.3 eV and *ω_s_* = 4.5 eV.

Hybridization of the sphere and the cavity results in two fundamental frequencies, *ω*_±_, as explained above. Nanopillars are treated as particles that are elongated in one (z) direction perpendicular to that of the light polarization vector. When spherical particles deform toward elongated spheroids, the plasmon frequency changes and become dependent upon the orientation of the elongated spheroid. For an Ag nanoshell with inner and outer radii of 60 and 65 nm one can compute the bonding and antibonding frequencies. The bonding and antibonding frequencies are 1.78 eV (697 nm) and 7.6 eV (163 nm), respectively. These frequencies are in good agreement with our experimental data since the angle of incidence is normal to the substrate and surface of the pillars. The electric field is perpendicular to the long axis of the pillar so the longitudinal component of the surface plasmon which corresponds to the length of the pillars is not excited; however, changing the angle of incidence can excite it and depending on the polarization of light, this causes a red or blue shift in the SPR frequency.

In a simple harmonic oscillator model, the plasma frequency is proportional to the restoring force between the polarized charges. When the electric field is parallel to the long axis of the pillars (*p* polarization), induced surface charges are generated at the top and bottom of the pillars. While the charge density is low, the separation between opposite charges is large (long axis of the pillars), which reduces the restoring force and consequently reduces the plasma frequency. The situation for transverse polarization (*s*) is reversed and results in a blue shift in the plasma frequency [62].

### 2.6. Hybrid Metal and Metal Oxide Core Shell Nanostructured Arrays

**Figure 6:**
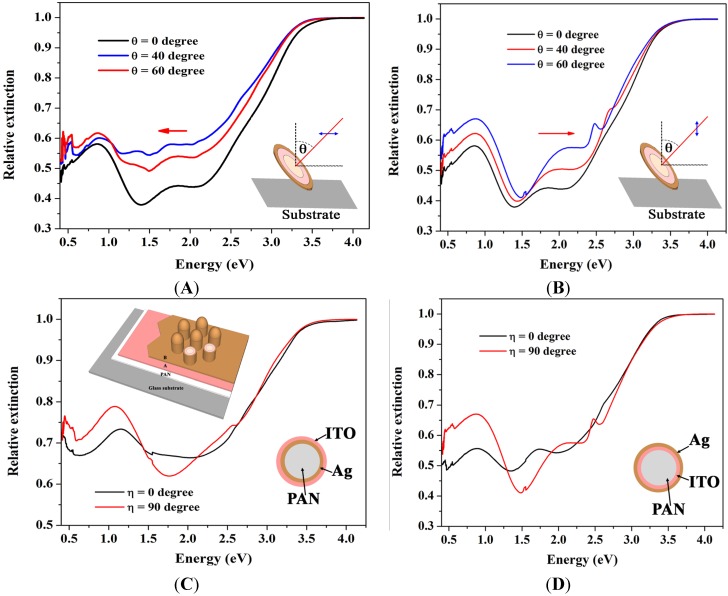
Relative extinction of ITO/Ag multilayered pillars at different angles of incidence for incident polarizations (**A**) parallel to the substrate (**B**) perpendicular to substrate and of (**C**) Ag/ITO and (**D**) ITO/Ag multilayered nanopillars at different polarizations of incident light for 60° angle of incidence. Bonding and antibonding hybridized plasmons are more separated at transverse (90°) polarization. Inset in A and B are schematic descriptions of incident angles and polarizations with respect to the pillars and substrate surface.

An ITO/Ag core shell nanopillar structure is investigated using the same strategy. 130 nm ITO is deposited on the PAN pillars followed by deposition of 10 nm of Ag; note that the ITO coating is not conformal around and between the pillars. The core shell particle can be separated into cavity (ITO core) and solid prolate spheroid (Ag). The plasma frequencies of the particle and cavity for a spherical particle are given by [62]

(5) $$\omega_{s}=\omega_{B}\sqrt{l/\left(\varepsilon_{E}\left(l+1\right)+\varepsilon_{s}l\right)}\;\text{and}\;\omega_{c}=\omega_{B}\sqrt{\left(l+1\right)/\left(\varepsilon_{s}\left(l+1\right)+\varepsilon_{c}l\right)}$$

where the *s*, *B*, *E*, and *c* subscripts refer to particle, bulk, embedding medium, and cavity properties, respectively, in a spherical geometry. If we assume a thin metal nanoshell in vacuum, then the equation reduces to the well-known Mie theory plasmon frequencies mentioned earlier. However in a cylindrical geometry, solutions to the Laplace equation are associated Legendre functions, which are functions of the aspect ratio of the pillar. In ITO\Ag coreshell pillars, if we assume *ε_c_* = 4 (dielectric constant of ITO), *ω_s_* and *ω_c_* are 3.083 eV (402 nm) and 2.57 eV (483 nm), respectively. The tunable hybridized frequencies arise from interaction between sphere and cavity frequencies. The strength of the interaction determines the frequencies of the hybridized plasmons that can be controlled by the thickness of the layers. Lower and higher frequency plasmons are dominated by sphere and cavity plasmons, respectively [63]. The extinction coefficient spectra of ITO/Ag core shell pillars at various angles of incidence for zero and 90 degree polarizations are shown in Figure 6.

Upon illumination of the pillar at an angle different from normal incidence, an ellipsoid cross section of the pillar will be excited. A schematic of the ellipsoid is shown in the Figure 6 inset. With increasing angle of incidence, once the light polarization is parallel to the long axis of the ellipsoid due to low density of polarized charges and longer distance between them, the energy of the plasmon is reduced and there is a red shift in the SPR frequency compared to normal incidence as it can be seen in Figure 6A; however, for incident light with opposite polarization, polarized charges will be formed along the minor axis and cause a blue shift in the plasmon frequency as shown in Figure 6B.

The dielectric constant of the core influences the anti-bonding plasma frequency. The dielectric constant of ITO is much higher than that of PAN. Dielectric charges are induced on the inner surface of the Ag nanoshell. Polarized charges will reduce the effective oscillating surface charges on the Ag nanoshell which, in turn, cause a red shift in the SPR frequency. Since the Ag shell around the pillars is very thin compared to the ITO core, the interaction between sphere and cavity plasmons is strong and a strong red shift in the bonding and antibonding hybridized frequencies is observed. It is interesting to note that the extinction spectra in Figure 6A,B, are slightly different in the case of normal incidence (θ = 0 degree). These graphs should be the same for a symmetric pillar since they are different only by polarization. A possible origin of this difference could be from a collective SPP oscillations of the 2D nanopillar array. SPPs are known to couple laterally through their optical near fields, giving rise to collective surface plasmon effects. Using the dispersion relation of such collective surface plasmons, it has been shown that propagation of SPPs in 2D arrays of nanorod and nanopillars, allows for polarization conversion and optical filtering [64]. In systems with two-dimensional (2D) arrays of metal-semiconductor multilayer nanopillars arranged in the x-y plane, as used here (see Figure 1A), the large optical anisotropy makes the optical characteristics sensitively dependent on the angle of incidence and polarization state of the incoming light [65,66,67].

The extinction coefficients of several core shell structures at 60° angle of incidence for two polarizations are plotted in Figure 6C,D. Since at this angle the plasmon mode along the longer axis of the pillar is excited, the bonding frequency of the hybridized plasmon is more pronounced. The higher frequency (antibonding) plasmon doesn’t change its intensity with polarization. However, there is a significant polarization-dependent change in intensity for the lower frequency (bonding) plasmon. For transverse (90°) polarization, the bonding frequency has a higher intensity than for parallel polarization. In this case bonding and antibonding frequencies are more separated.

**Figure 7:**
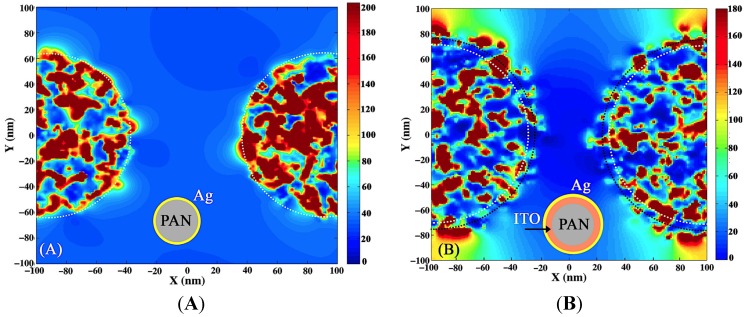
xy distribution of the electric field component of light at the bonding frequency of the Ag nanoshell with (**A**) PAN (**B**) ITO cores.

Increasing the dielectric constant of either core or embedding media results in a strong red shift of both symmetric and antisymmetric dipole plasmon modes, however it affects them in different ways. As the dielectric constant of the core increases from PAN to ITO in PAN/Ag and PAN/ITO/Ag, respectively, the antisymmetric plasma frequency undergoes a larger red shift than the symmetric frequency. The ratio of the antisymmetric mode intensity to the symmetric mode increases, as can be seen in the simulation shown in Figure 7. The situation for Ag/ITO nanopillar arrays is reversed; in this case a larger red shift is obtained in the symmetric plasmon mode. The relative intensity of the symmetric to the antisymmetric mode increases with increasing embedding dielectric constant. A larger dielectric constant for the embedding medium induces larger differences between sphere and cavity plasmon frequencies due to the lower sphere frequency, which results in a strong contribution of the sphere plasmon to the symmetric hybridized frequency [68].

Symmetric core shell structures were fabricated by depositing a 10 nm of Ag sandwiched between two ITO layers. The dielectric constants of the top ITO layer were calculated using the Bruggemann mixing law [37]. Here we assume that the ITO pillars are in the form of inclusions inside air. From the experimental transmission spectrum of the sample, we see a maximum in the extinction coefficeint at 1.07 eV. There is also a local maximum around 1.82 eV, which is very similar to the bonding hybridized plasmon of Ag/PAN nanopillars. In a heterostructure, dipole and quadrupole moments of the pillars are different, however in this geometry, since the dielectric environments are the same on both sides of the Ag, the net dipole moment is zero and dipoles can’t be excited by light; therefore the antibonding plasma frequency vanishes while the bonding frequency is still observable. This can be seen in the absorption spectrum of the symmetric structure as the high frequency plasmon mode which is shorter than 690 nm doesn’t exist, which is not the case for asymmetric core shell electrodes.

## 3. FDTD Simulation

The finite difference time domain (FDTD) method is used for simulating the field distribution around the pillars. Periodic boundary conditions in the x and y directions and a perfectly matched layer (PML) in the z direction are applied. Incident light is polarized along x(y) direction and propagates in the z direction as shown in Figure 7. The intensity is averaged over x and y directions of polarization and the electric field distribution around PAN pillars coated with random sized Ag particles is shown in Figure 7 at bonding plasmon wavelengths (690 nm for PAN/Ag and 1150 nm for ITO/Ag). In all simulations, multiple thicknesses, close (±10 nm) to experimental thickness of ITO and Ag, were tested. Within this range of thicknesses no significant differences in the simulation results were observed.

**Figure 8:**
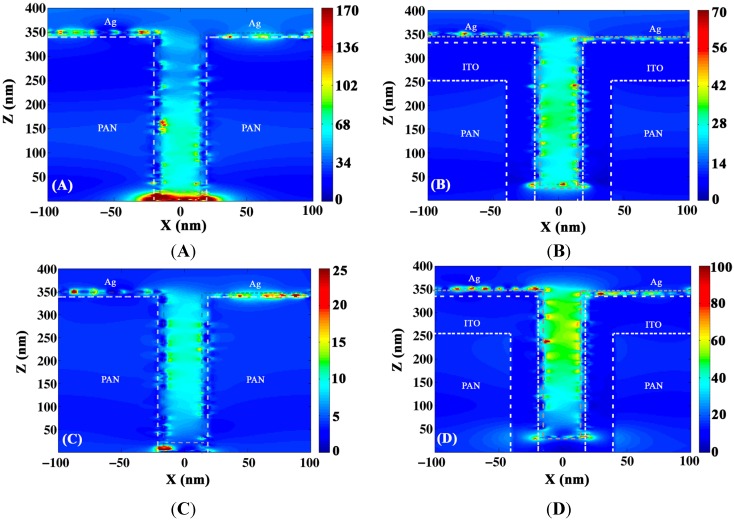
Comparison of electric field intensity in PAN/Ag and ITO/Ag nanoshells at 690 nm (**A** and **B**) and 1150 nm (**C** and **D**).

Figure 8 shows the xz profile of the electric field intensity of light at the two resonance wavelengths 690 nm and 1150 nm. As one can see in the PAN/ITO/Ag core shell the surface plasmons are less localized and extend over the distance between the pillars compared to PAN/Ag, which supports more localized surface plasmons. It is important to note that simulations suggest that a significantly reduced diameter would lead to a blue shift whereas an increased diameter would lead to a red shift in the SPP resonance.

**Figure 9:**
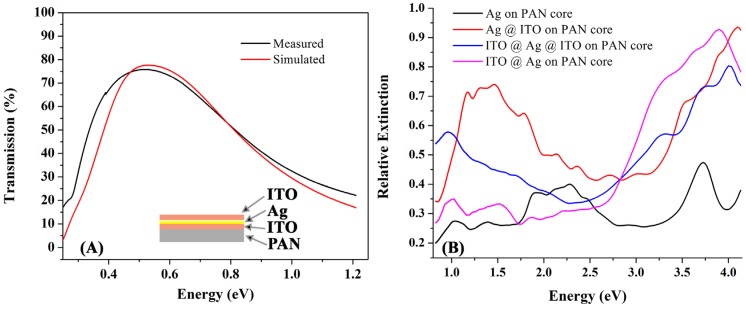
(**A**) Simulated and experimental transmission spectra of planar ITO/Ag/ITO multilayer structure showing excellent agreement. The mismatch in the shorter wavelength region might be due to sample nonuniformity. (**B**) Simulated extinction coefficient spectra of various nanostructure arrays. The spectra follow the same trend as the experimental data in Figure 2.

The dielectric constants of the different materials have been experimentally measured using spectroscopic ellipsometry. Figure 9 shows the simulated and experimental transmission spectra of a simulated and measured planar ITO/Ag/ITO sample. The broadening in the experimental data is due to inhomogeneities in the sample, but they are still in excellent agreement.

Maxwell equations solutions in cylindrical coordinates with appropriate boundary conditions are modified Bessel and Hankel functions, depending on the material properties in the core and shell layers [69]. Tangential components of the electric field and the magnetic field must be continuous at the interface.

The charge density distribution amplitude around the pillars for 50 nm Ag coated on the pillars was simulated using FDTD. As mentioned above, deposition of materials on the pillars does not generally result in conformal coating. In this case the sidewalls of the pillars are coated with 15 nm Ag while the thickness of Ag on top of the pillars is 50 nm.

**Figure 10:**
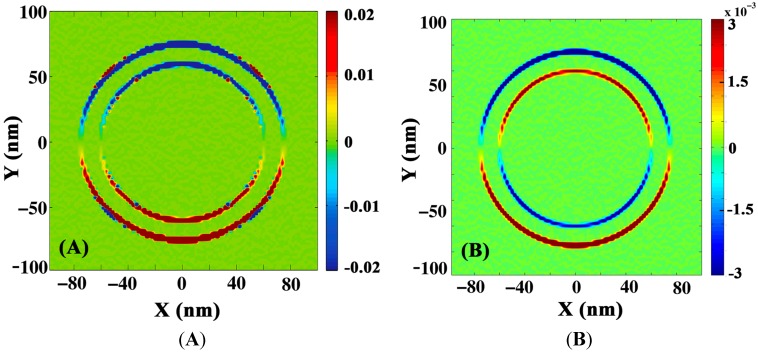
Simulated 2D distribution of charge density for (**A**) bonding and (**B**) antibonding hybridized plasmon frequency.

Figure 10 indicates that, at lower frequencies, which correspond to a bonding hybridized plasmon, the distribution of charge carriers around the pillars has a symmetric profile in contrast to the higher antibonding frequency which leads to an anti-symmetric profile. In core shell structures, cavity and spherical plasmons induce primary charges on both surfaces of the metal. Each plasmon also generates secondary charges on the other side of the interface. The interaction between the plasmons is written as the Coulomb interaction between induced charges. In a symmetric alignment of polarized charges, the Coulomb potential is repulsive, since the charges are of the same polarity. However, secondary induced charges attract each other. The attractive interaction is larger than the repulsive potential and favors a symmetric alignment of the charges with a lower energy. For an antisymmetric distribution of charges, however, the net potential is repulsive and corresponds to a higher frequency resonance. Furthermore, since only dipole moments are excited by light, there are two nodes around the circumference of the nanopillars. According to the plasmon hybridization model, the number of nodes along the circumference is 2*l*, where *l* is the multipolar order.

**Figure 11:**
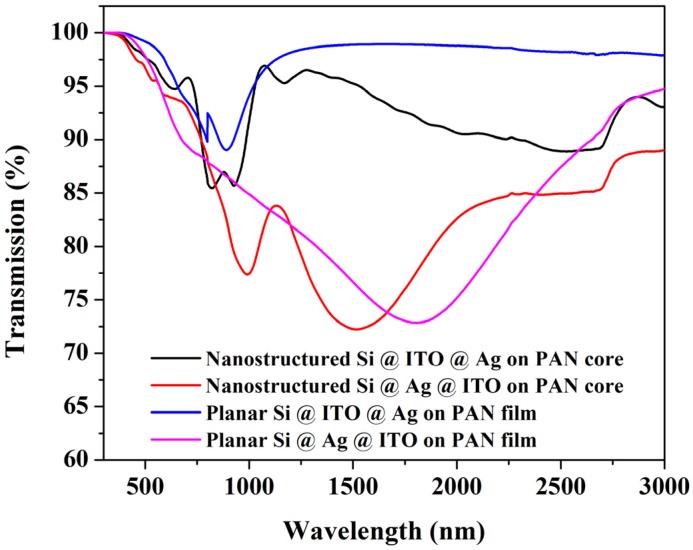
Transmission spectra of different sets of Si coated nanopillar structures.

These experiments and FDTD simulations indicate that following the standard plasmon hybridization model, and using a multilayer asymmetric configuration Ag@ITO on PAN nanopillars, a broadband nonlocal plasmon resonance can be achieved. To further prove potential efficacy of these structures in a Si photodiode type scenario, several structures were fabricated by depositing Si on Ag@ITO and ITO@Ag nanostructures and were compared with the corresponding planar family. Figure 11 compares the transmission spectra of silicon coated structures, where 50 nm thick Si was deposited on 10 nm thick Ag coated on 130 nm thick layer of ITO or vice versa. Nanostructured Ag@ITO on PAN nanopillars shows significant improvement in absorption over its planar counterpart and a broadband response from 1 to 1.6 microns. Another advantage of adding a silicon or a doped silicon layer is to further modify the resistivity of the underlying nanostructures. As indicated in Table 1, one of the drawbacks of the high surface area nanoarchitectures is the increased resistivity in the metal semiconductor multilayer structures. Whereas the tunable surface plasmons and enhanced surface area help significantly in improving the detector efficiency, most importantly in the NIR region otherwise inaccessible to silicon detectors, increased resistivity could lead to potentially-reduced sensitivity and wavelength responsivity. This is a potential tradeoff that must be optimized for a satisfactory performance of a detector in these nanoarchitectured systems. The detector structure developed here uses a nanostructure with much larger pitch than the ones reported in Table 1, enabling better conductivity and reduced resistivity. Tuning pitch dimensions in these structures would be key to optimizing the sensitivity tradeoff.

## 4. Conclusions

In summary, we have demonstrated a broadband plasmonic structure close to a working Schottky-type Si photodiode structure with improved absorption within the NIR wavelengths of interest for telecommunication. This approach can clearly be applied to enhancing the detection sensitivity of a variety of detectors in the near-infrared wavelength region, highlighting the benefits of combining conventional plasmonic materials, such as Ag, with unconventional plasmonic materials such as ITO.
